# Different types of working after retirement on the changes in cognitive function among taiwanese retirees: 3-year follow-up study

**DOI:** 10.1186/s12889-025-25325-0

**Published:** 2025-12-05

**Authors:** Tai-Kang Wu, Ching-Ju Chiu, Li-Jung Elizabeth Ku, Jer-Hao Chang, Susan C. Hu

**Affiliations:** 1 Department of Public Health, College of Medicine, National Cheng Kung University, Tainan, 70101 Taiwan; 2 Institute of Gerontology, College of Medicine, National Cheng Kung University, Tainan, 70101 Taiwan; 3 Department of Occupational Therapy, College of Medicine, National Cheng Kung University, Tainan, 70101 Taiwan; 4 Department of Healthcare Administration, College of Medical and Health Sciences, Asia University, Taichung, 41354 Taiwan

**Keywords:** Retirement, Education, Working after retirement, Retirees, Aging, Cognitive function

## Abstract

**Objective:**

This study aimed to examine the associations between working after retirement and three-year changes in cognitive function, focusing on differences by work type, work hours, and education level in Taiwan.

**Methods:**

Data were drawn from the Taiwan Health and Retirement Study (THRS), a nationally representative survey of labor and civil service pension recipients aged 50–74 years. A total of 2,176 retirees with valid cognitive assessments across two waves (2015–2016 and 2018–2019) were included in the analysis. Cognitive function was measured by the Saint Louis University Mental Status (SLUMS) examination. Working after retirement was categorized along two axes: type (paid, unpaid, self-employment, vs. fully retired) and hours (full-time, part-time, vs. fully retired). Multiple linear regression models were used to examine associations with three-year cognitive change, with stratified analyses by education level.

**Results:**

In the overall sample, neither classification of working after retirement was significantly associated with cognitive change. However, among retirees with lower educational attainment, those engaged in full-time work experienced a slower decline in cognition compared with those who were fully retired (β = 1.04, SE = 0.50, *p* = .036). No such association was observed among those with higher education.

**Conclusion:**

These findings highlight heterogeneity in the associations between working after retirement and cognitive aging, with potential benefits of full-time work concentrated among lower-educated retirees. However, the lack of consistent effects across sensitivity analyses indicates the need for cautious interpretation. Future research should employ longer follow-ups and causal inference methods to better clarify the mechanisms. Policies should recognize that maintaining cognitive health requires not only employment opportunities but also broader support for diverse forms of engagement, such as social participation, across the lifespan.

**Supplementary Information:**

The online version contains supplementary material available at 10.1186/s12889-025-25325-0.

## Introduction

The rapid aging of the global population has elevated the study of retirees’ cognitive health to a pressing public health priority. According to the United Nations World Population Prospects [37], declining fertility rates drive a demographic shift, with individuals aged 65 and older expected to outnumber those under 18 by the late 2070s. This transition poses significant challenges to labor markets, healthcare systems, and social welfare structures worldwide. Taiwan exemplifies this trend; by July 2025, its aging population had already reached 19.64%, continuing its progression toward a super-aged society [21]. This accelerated aging underscores the importance of examining labor participation and cognitive health—key factors that impact the well-being of older adults.

Retirement, a major life transition, may diminish cognitive stimulation, potentially accelerating decline—a notion aligned with the "use it or lose it" hypothesis. However, the evidence regarding the cognitive benefits of post-retirement work remains mixed and inconclusive. While some observational studies have linked post-retirement work to improved cognitive outcomes, others report null or even negative findings [19, 46]. These discrepancies suggest the relationship is not straightforward and warrants a more nuanced investigation.

A central challenge in interpreting these findings is the difficulty of separating correlation from causation. The decision to continue working after retirement is not random; individuals with better health and higher cognitive function are more likely to remain employed [35], creating a substantial risk of selection bias. While most studies are associative, a small number of quasi-experimental analyses have attempted to better estimate causal effects, with similarly mixed conclusions. For instance, regression discontinuity analyses of U.S. cohorts suggest that retirement accelerates cognitive decline [8]. In contrast, an instrumental variable study in Japan found no significant causal effect of full retirement but did identify negative effects of continued part-time work with the same employer [23]. This scarcity of consistent causal evidence underscores the need for studies that, while observational, carefully account for heterogeneity in work and retiree characteristics.

One primary reason for inconsistent results is that “working after retirement” is often treated as a monolithic concept. To address this, we conceptualize its heterogeneity along two complementary axes: remuneration status (paid, unpaid, and self-employment) and time commitment (full-time vs. part-time). Remuneration reflects role characteristics such as organizational structure and task complexity, while time commitment indexes the intensity and duration of cognitive engagement. Collapsing across either dimension risks obscuring meaningful variation—for example, high-demand unpaid caregiving differs substantially from flexible paid part-time work. This two-way classification enhances construct validity, facilitates tests of moderation by individual characteristics, and yields policy-relevant contrasts.

Beyond the heterogeneity of work, associations may also be moderated by retirees’ characteristics. The Cognitive Reserve Theory offers a valuable framework, positing that lifelong experiences, such as education, build resilience against age-related decline [10, 32]. A systematic review and meta-analysis by Seblova et al. [30] found robust evidence linking education with baseline cognitive performance but no significant effect on the rate of cognitive change over time. In contrast, recent evidence suggests that individuals with lower education are more susceptible to faster cognitive decline, with retirement further intensifying this process [1]. Taken together, these inconsistent findings suggest that the moderating role of education in cognitive change remains unsettled and warrants further empirical testing.

The literature on retirement and cognition is heavily skewed toward Western contexts, whereas evidence from Asia reveals substantial heterogeneity. For instance, post-retirement work was associated with delayed cognitive decline in Japanese men [27], showed no significant association in Korea [13], and was linked to deterioration among rural Chinese retirees with access to pensions [24]. These divergent findings highlight the importance of local institutional and policy contexts. In Taiwan, retirement protection is primarily organized through occupation-based schemes, with most private-sector employees covered by the Labor Insurance system and most public employees covered by the Civil Servants Insurance system. Under labor insurance, voluntary retirement is possible if individuals meet one of the following conditions: age 55 or older with 15 or more years of service, 25 or more years of service regardless of age, or age 60 or older with 10 or more years of service, while the statutory retirement age is 65. In the public sector, the statutory retirement age is also 65; however, civil servants may qualify for early retirement at age 55 or older with at least 25 years of service, or with at least 20 years of service in hazardous or special posts. Notably, both systems allow retirees to continue working while receiving pension benefits. However, financial necessity is more common among labor insurance retirees due to relatively modest benefits, whereas civil servants generally face less economic pressure because of higher pension replacement rates. However, civil servants who re-enter public institutions remain subject to a minimum-wage threshold: when reemployment earnings exceed this threshold, monthly pension payments are typically suspended or offset [12, 43]. This institutional context makes Taiwan a distinctive setting for examining the relationship between post-retirement work and cognitive aging.

This study is guided by the “use it or lose it” hypothesis [29, 38], the “cognitive reserve theory” [10, 32], and the ecological framework of engagement–cognition interaction [34], which emphasize context-driven strategies for maintaining cognition. Using longitudinal data from the Taiwan Health and Retirement Study (THRS), we examine the associations between different types of working after retirement and three-year changes in cognitive function, with particular attention to whether educational attainment moderates these associations within Taiwan’s institutional context.

## Methods

### Participants

This study used data from the Taiwan Health and Retirement Study (THRS), a nationally representative survey of retirees aged 50–74 years who were covered by Taiwan’s Labor Insurance (for private-sector employees) or Civil Servants Insurance (for public employees). The THRS was commissioned by the Health Promotion Administration, Ministry of Health and Welfare, and conducted by the Center for Health Cities Research at National Cheng Kung University.

The baseline survey (2015–2016) included 3,141 valid respondents, sampled through a multistage stratified design across 53 townships and districts to ensure proportional representation by geographic region and urbanization level. The follow-up survey (2018–2019) successfully re-interviewed 2,448 individuals, after accounting for 85 deaths, corresponding to a response rate of 80.1%.

To ensure valid assessment of cognitive change and minimize measurement error, we excluded participants who: (1) missed two or more items on the Saint Louis University Mental Status Examination (SLUMS) in either wave (*n* = 41); (2) SLUMS scored extremely low at baseline (< 6.88 points, > 3 SD below the mean; *n* = 42); (3) reported no employment history prior to retirement (*n* = 47); (4) had missing information on working after retirement (*n* = 3); or (5) reported functional impairment in daily activities or had missing responses to the relevant items (*n* = 37). In addition, respondents with missing values on covariates used in the regression models (*n* = 102) were excluded. After applying these criteria, the final analytic sample comprised 2,176 retirees.

## Measurement

### Dependent variable

This study used the Saint Louis University Mental Status (SLUMS) scale as a comprehensive cognitive assessment tool. Unlike the Mini-Mental State Examination (MMSE), SLUMS demonstrates higher sensitivity and specificity in detecting mild cognitive impairment [2, 36]. Chinese versions of SLUMS have been validated in Taiwan, demonstrating robust reliability and validity across diverse educational levels [41, 44]. The SLUMS is an 11-item scale assessing multiple domains, including orientation, memory, attention, and executive function, with scores ranging from 0 to 30.

The primary outcome of this study was the three-year change in cognitive function. This was operationalized by creating a continuous change score, calculated as the SLUMS score at follow-up (wave 2) minus the SLUMS score at baseline (wave 1). While clinical cutoffs for SLUMS scores vary by education level, our analysis utilized the raw, continuous scores to capture the full spectrum of cognitive change and avoid information loss from categorization.

### Independent variables

Independent variables were categorized into three main groups: types of work after retirement, pre-retirement occupation, and early retirement.

- Types of working after retirement
  In this study, retirement was defined as the commencement of receiving pension benefits from the Labor Insurance or Civil Servants system. Working after retirement was assessed at baseline (Wave 1, 2015–2016) with the question, “What is your current retirement status?” The detailed questionnaire items and classification criteria, consistent with prior research [39], are provided in Supplementary Table S1. For analysis, working after retirement was categorized along two dimensions. Classification I (by work type) included: (1) fully retired, (2) paid work, (3) unpaid work, and (4) self-employment. Classification II (by work hours) comprised: (1) fully retired, (2) full-time work, and (3) part-time work. This dual framework enables a more nuanced examination of working patterns after retirement.
- Pre-retirement occupation
  Participants were asked, “What was your occupation before you retired?” The responses were initially classified into five categories based on the Standard Occupational Classification of the Republic of China: (1) unskilled, (2) semi-skilled, (3) skilled, (4) professionals, and (5) higher-level professionals and managers. This system is consistent with the International Standard Classification of Occupations (ISCO-08), with localized adjustments to reflect Taiwan’s labor market structure. For analytical parsimony and to address limited sample sizes, these five categories were consolidated into three groups: (1) unskilled, (2) semi-skilled/skilled, and (3) managerial/professional. This grouping approach aligns with prior research [39, 40]. The detailed questionnaire items used in this measure are provided in Supplementary Table S2.
- Early retirement
  Early retirement status was assessed by asking participants, "What was the reason for your first retirement?" Response options included institutional factors, such as reaching retirement age, meeting eligibility criteria for preferential retirement, and qualifying for a monthly pension, as well as personal factors, including health issues, family obligations, and other reasons. Participants who selected "reaching retirement age" were categorized as (1) No (not early retirement), while those who chose other options were categorized as (2) Yes (early retirement).

### Covariates

All covariates were measured at baseline (Wave 1, 2015–2016). To reduce confounding, we adjusted for sociodemographic, health, lifestyle, and social engagement factors that have been identified in prior research as predictors of late-life cognition. Sociodemographic variables included age, gender (male/female), educational attainment (primary school or lower, junior/high school, college or higher), marital status (single or married), years since retirement (0–5, 6–9, ≥ 10 years), and perceived economic status (difficult vs. not difficult/fair). Health-related factors comprised self-rated health (poor, fair, or good/excellent), depression (no/yes), hypertension (no/yes), stroke (no/yes), and diabetes (no/yes). Lifestyle factors included regular exercise (yes/no), smoking (yes/no), and alcohol consumption (yes/no). Finally, social participation was categorized as none, membership in one club/organization, or membership in two or more clubs/organizations.

### Statistical analysis

Descriptive statistics were used to summarize demographic factors, pre-retirement work characteristics, and working after retirement. Group differences were examined using chi-square tests for categorical variables and t-tests for continuous variables. Associations between individual predictors and three-year cognitive change scores (follow-up minus baseline SLUMS) were further assessed in bivariate analyses.

Before model estimation, multicollinearity was examined using variance inflation factors (VIFs). All values ranged from 1.03 to 1.70, well below the commonly cited threshold of 5 that indicates potential concern [15]. Multivariable linear regression models were then estimated to assess the associations of demographic, pre-retirement, and post-retirement work characteristics with cognitive change scores. Analyses were based on 2,176 participants with complete data on all covariates.

Given prior evidence that educational attainment shapes lifetime occupational opportunities, exposure to cognitively demanding or hazardous work, and access to cognitively stimulating environments, we treated education as a potential moderator in the association between post-retirement work and cognitive change. Education occurs prior to labor market entry, providing a stable marker of cognitive reserve across the life course. Accordingly, we conducted stratified regressions by education level (lower vs. higher) to examine whether the cognitive implications of post-retirement work differed across educational groups.

To evaluate robustness, sensitivity analyses were performed by (i) excluding early retirees and (ii) testing alternative specifications of working after retirement (binary indicator and linear dose variable). Finally, stratified regressions by gender were conducted to examine potential heterogeneity.

## Results

Descriptive statistics are based on the analytical sample (*n* = 2,176) to align with regression analyses. Table 1 summarizes baseline characteristics by retirement status. Retirees who continued to work were younger, more likely to be male, and had lower educational attainment compared with those who were fully retired. Overall, 52.1% were aged 50–64, 31.2% had completed only primary school or less, and 33.7% held a college degree or higher. Most were married (83.5%), and 73.5% reported stable economic status. Additional comparisons by gender are provided in Supplementary Table S3, showing marked sex differences in most sociodemographic and health variables.

**Table 1:** Baseline Characteristics of Study Participants by Working after Retirement (*n* = 2,176)

Variables	Categories	Total (*n* = 2,176)	Not Working (*n* = 1,599)	Working (*n* = 577)	*p*-value
Gender	Male	1,219 (56.0)	884 (55.3)	335 (58.1)	.250
	Female	957 (44.0)	715 (44.7)	242 (41.9)	
Age	50–64	1,134 (52.1)	776 (48.5)	358 (62.0)	** <.001**
	65–74	1,042 (47.9)	823 (51.5)	219 (38.0)	
Education	Primary school or less	678 (31.2)	489 (30.6)	189 (32.8)	** <.001**
	Junior or high school	764 (35.1)	533 (33.3)	231 (40.0)	
	College or more	734 (33.7)	577 (36.1)	157 (27.2)	
Marital status	Single	359 (16.5)	258 (16.1)	101 (17.5)	.448
	Married	1,817 (83.5)	1,341 (83.9)	476 (82.5)	
Economic status	Not difficult/even	1,599 (73.5)	1,194 (74.7)	405 (70.2)	**.037**
	Difficult	577 (26.5)	405 (25.3)	172 (29.8)	
Years after retirement	0–5 years	881 (40.5)	625 (39.1)	256 (44.4)	**.016**
	6–9 years	496 (22.8)	359 (22.5)	137 (23.7)	
	More than 10 years	799 (36.7)	615 (38.5)	184 (31.9)	
Self-rated health	Poor/not good	291 (13.4)	226 (14.1)	65 (11.3)	.085
	Fair	934 (42.9)	667 (41.7)	267 (46.3)	
	Excellent/good	951 (43.7)	706 (44.2)	245 (42.5)	
Depression	No	1,950 (89.6)	1,422 (88.9)	528 (91.5)	.082
	Yes	226 (10.4)	177 (11.1)	49 (8.5)	
Hypertension	No	1,365 (62.7)	1,007 (63.0)	358 (62.1)	.692
	Yes	811 (37.3)	592 (37.0)	219 (38.0)	
Stroke	No	2,139 (98.3)	1,572 (98.3)	567 (98.3)	.943
	Yes	37 (1.7)	27 (1.7)	10 (1.7)	
Diabetes	No	1,780 (81.8)	1,297 (81.1)	483 (83.7)	.166
	Yes	396 (18.2)	302 (18.9)	94 (16.3)	
Physical activity	No	959 (44.1)	624 (39.0)	335 (58.1)	** <.001**
	Yes	1,217 (55.9)	975 (61.0)	242 (41.9)	
Smoking	No	1,822 (83.8)	1,357 (84.9)	465 (80.6)	**.017**
	Yes	354 (16.3)	242 (15.1)	112 (19.4)	
Alcohol consumption	No	1,817 (83.5)	1,343 (84.0)	474 (82.1)	.307
	Yes	359 (16.5)	256 (16.0)	103 (17.9)	
Social participation	No	1,386 (63.7)	1,020 (63.8)	366 (63.4)	.987
	1 organization	512 (23.5)	375 (23.5)	137 (23.7)	
	≥ 2 organizations	278 (12.8)	204 (12.8)	74 (12.8)	
Pre-retirement occupations	Unskilled	410 (18.8)	304 (19.0)	106 (18.4)	**.014**
	Semi-skilled/skilled	1,215 (55.9)	866 (54.2)	349 (60.5)	
	Managers/professionals	551 (25.3)	429 (26.8)	122 (21.1)	
Early retirement	No	1,284 (59.0)	939 (58.7)	345 (59.8)	.655
	Yes	892 (41.0)	660 (41.3)	232 (40.2)	
Baseline SLUMS Score, mean (SD)		23.29 (4.66)	23.20 (4.67)	23.56 (4.64)	.103
SLUMS Change Score, mean (SD)		− 0.34 (4.64)	− 0.33 (4.66)	− 0.38 (4.59)	.798

Table 2 presents mean changes in SLUMS scores across subgroups. Greater cognitive decline was observed among older participants, those with lower education, unmarried retirees, and those reporting poorer health, hypertension, or limited social participation. Cognitive trajectories significantly differed by pre-retirement occupational categories. Retirees from lower-complexity occupations showed the most significant decline in SLUMS scores over the three years (mean change = −0.67 ± 5.31, *p* < 0.001), compared with those from semi-skilled/skilled (−0.27 ± 4.45, *p* = 0.083) and managers/professionals (−0.12 ± 4.13, *p* = 0.432). In contrast, neither classification of working after retirement (by type or hours) was significantly related to cognitive change in unadjusted analyses.

**Table 2:** Bivariate analysis of the changes in cognitive function and related factors (*n* = 2,176)

			**SLUMS Score**			**Paired-t**
**Variables**	**Categories**	**n**	**Baseline**	**Follow-up**	**Difference**	***p*-value**
Gender	Male	1,219	23.65 ± 4.40	23.16 ± 4.73	−0.49 ± 4.49	** <.001**
	Female	957	22.83 ± 4.95	22.68 ± 5.24	−0.15 ± 4.82	.285
Age	50–64	1,134	24.16 ± 4.39	24.02 ± 4.41	−0.14 ± 4.44	.283
	65–74	1,042	22.35 ± 4.77	21.79 ± 5.28	−0.56 ± 4.84	** <.001**
Education	Primary school or less	678	20.32 ± 4.71	19.66 ± 5.37	−0.67 ± 5.31	** <.001**
	Junior or high school	764	23.85 ± 4.02	23.58 ± 4.10	−0.27 ± 4.45	.083
	College or more	734	25.46 ± 3.75	25.34 ± 3.60	−0.12 ± 4.13	.432
Marital status	Single	359	22.22 ± 4.96	21.82 ± 5.59	−0.40 ± 4.74	.104
	Married	1,817	23.51 ± 4.57	23.17 ± 4.81	−0.33 ± 4.62	**.002**
Economic status	Not difficult/even	1,599	23.73 ± 4.51	23.42 ± 4.59	−0.31 ± 4.51	**.006**
	Difficult	577	22.10 ± 4.86	21.66 ± 5.69	−0.44 ± 4.98	**.007**
Years after retirement	0–5 years	881	23.56 ± 4.55	23.12 ± 4.82	−0.25 ± 4.64	**.048**
	6–9 years	496	22.92 ± 4.86	22.81 ± 5.11	−0.11 ± 4.50	.754
	More than 10 years	799	23.23 ± 4.65	22.85 ± 5.05	−0.39 ± 4.68	**.020**
Self-rated health	Poor/not good	291	21.33 ± 5.21	21.30 ± 5.71	−0.03 ± 5.03	.926
	Fair	934	22.96 ± 4.90	22.60 ± 5.18	−0.36 ± 4.61	**.016**
	Excellent/good	951	24.12 ± 4.33	23.70 ± 4.68	−0.42 ± 4.56	**.004**
Depression	No	1,950	23.56 ± 4.46	23.25 ± 4.77	−0.31 ± 4.69	**.020**
	Yes	226	22.31 ± 4.96	21.74 ± 5.39	−0.57 ± 4.78	**.017**
Hypertension	No	1,365	23.33 ± 4.64	23.01 ± 5.03	−0.32 ± 4.64	**.003**
	Yes	811	23.10 ± 4.78	22.65 ± 4.61	−0.44 ± 4.62	**.049**
Stroke	No	2,139	23.32 ± 4.70	23.01 ± 5.02	−0.31 ± 4.63	**.002**
	Yes	37	22.40 ± 4.95	21.79 ± 5.36	−0.61 ± 4.69	**.010**
Diabetes	No	1,780	23.52 ± 4.54	23.14 ± 4.84	−0.38 ± 4.59	**.001**
	Yes	396	21.33 ± 5.21	21.30 ± 5.71	−0.03 ± 5.03	.926
Physical activity	No	959	23.08 ± 4.75	22.57 ± 5.13	−0.50 ± 4.64	**.002**
	Yes	1,217	23.42 ± 4.59	23.16 ± 4.85	−0.26 ± 4.73	**.048**
Smoking	No	1,822	23.42 ± 4.61	23.18 ± 4.86	−0.25 ± 4.64	**.048**
	Yes	354	22.96 ± 4.90	22.60 ± 5.18	−0.36 ± 4.61	**.016**
Alcohol consumption	No	1,817	23.14 ± 4.84	22.83 ± 5.05	−0.31 ± 4.64	**.010**
	Yes	359	23.20 ± 4.67	22.87 ± 5.05	−0.33 ± 4.66	**.010**
Social participation	No	1,386	22.96 ± 4.90	22.60 ± 5.18	−0.36 ± 4.61	**.016**
	1 organization	512	23.42 ± 4.59	23.16 ± 4.85	−0.26 ± 4.73	**.048**
	≥ 2 organizations	278	24.16 ± 4.39	24.02 ± 4.41	−0.14 ± 4.44	.283
Working after retirement (Classification I)	Fully retired	1,599	23.09 ± 4.75	22.80 ± 5.10	−0.29 ± 4.69	**.011**
	Paid work	378	23.53 ± 4.77	23.21 ± 4.86	−0.32 ± 4.60	.166
	Unpaid work	94	24.66 ± 4.05	24.23 ± 4.04	−0.43 ± 4.43	.330
	Self-employment	105	22.47 ± 4.63	22.05 ± 4.85	−0.43 ± 5.19	.389
Working after retirement (Classification II)	Fully retired	1,599	23.09 ± 4.75	22.80 ± 5.10	−0.29 ± 4.69	**.011**
	Full-time job	221	24.24 ± 4.14	23.71 ± 4.23	−0.52 ± 4.28	.062
	Part-time job	356	23.08 ± 4.92	22.83 ± 5.05	−0.25 ± 4.91	.318
Pre-retirement occupations	Unskilled	410	20.32 ± 4.71	19.66 ± 5.37	−0.67 ± 5.31	** <.001**
	Semi-skilled/skilled	1,215	23.85 ± 4.02	23.58 ± 4.10	−0.27 ± 4.45	.083
	Managers/professionals	551	25.46 ± 3.75	25.34 ± 3.60	−0.12 ± 4.13	.432
Early retirement	No	1,284	22.96 ± 4.90	22.60 ± 5.18	−0.36 ± 4.61	**.016**
	Yes	892	23.42 ± 4.59	23.16 ± 4.85	−0.26 ± 4.73	**.048**

Table 3 shows regression estimates of working after retirement on cognitive change. In the overall sample, neither Classification I (paid, unpaid, self-employed vs. fully retired) nor Classification II (full-time, part-time vs. fully retired) was significantly associated with cognitive change after full adjustment (R^2^ = 0.293).

**Table 3:** Association between Working after retirement and 3-Year Cognitive Change (*n* = 2176)

	**Model 1**			**Model 2**		
**Variables**	**β**	**(SE)**	***p*-value**	**β**	**(SE)**	***p*-value**
Working after retirement (Classification I) (ref: fully retired)						
Paid Work	0.17	(0.23)	.457	-	-	-
Unpaid Work	0.35	(0.42)	.406	-	-	-
Self-employment	−0.17	(0.40)	.674	-	-	-
Working after retirement (Classification II) (ref: fully retired)						
Full-time	-	-	-	0.23	(0.29)	.432
Part-time	-	-	-	0.09	(0.24)	.715
Covariates						
Baseline SLUMS Score	**−0.61**	**(0.02)**	** <.001**	**−0.61**	**(0.02)**	** <.001**
Age (years)	**−0.62**	**(0.09)**	** <.001**	**−0.62**	**(0.09)**	** <.001**
Gender (ref: male)						
Female	0.18	(0.20)	.382	0.18	(0.20)	.365
Education (ref: primary school or less)						
Junior or high school	**1.92**	**(0.23)**	** <.001**	**1.93**	**(0.23)**	** <.001**
College or more	**2.57**	**(0.29)**	** <.001**	**2.58**	**(0.29)**	** <.001**
Marital status (ref: single)						
Married	−0.30	(0.24)	.198	−0.29	(0.24)	.212
Economic status (ref: difficult)						
Not difficult	0.25	(0.20)	.222	0.25	(0.20)	.221
Years after retirement** (**ref: 0–5 years)						
6–9 years	**0.51**	**(0.23)**	**.027**	**0.51**	**(0.23)**	**.026**
More than 10 years	0.40	(0.22)	.073	0.40	(0.22)	.070
Self-rated health (ref: poor)						
Fair	0.36	(0.27)	.188	0.36	(0.27)	.192
Excellent	0.43	(0.29)	.139	0.42	(0.29)	.141
Depression (Yes vs. No)	−0.32	(0.29)	.285	−0.31	(0.29)	.290
Hypertension (Yes vs. No)	−0.03	(0.18)	.885	−0.03	(0.18)	.866
Stroke (Yes vs. No)	**−1.49**	**(0.66)**	**.025**	**−1.50**	**(0.66)**	**.024**
Diabetes (Yes vs. No)	−0.29	(0.23)	.209	−0.28	(0.23)	.222
Physical activity (Yes vs. No)	0.22	(0.18)	.207	0.23	(0.18)	.200
Smoking (Yes vs. No)	−0.02	(0.25)	.948	−0.02	(0.25)	.943
Alcohol consumption (Yes vs. No)	−0.29	(0.25)	.238	−0.29	(0.25)	.230
Social participation (ref: no)						
1 organization	**0.84**	**(0.21)**	** <.001**	**0.86**	**(0.21)**	** <.001**
≥ 2 organizations	**0.70**	**(0.27)**	**.009**	**0.70**	**(0.27)**	**.009**
Pre-retirement occupations (ref: unskilled)						
Semi-skilled/skilled	0.30	(0.24)	.206	0.29	(0.24)	.225
Managers/professionals	0.59	(0.33)	.073	0.58	(0.33)	.077
Early retirement (Yes vs. No)	0.35	(0.18)	.056	0.34	(0.18)	.059
R square		0.293			0.293	

*β* regression coefficient, *SE* Standard Error. The dependent variable is the 3-year change in SLUMS score. Models were fully adjusted for all listed covariates

Table 4 examines education as a moderator. When employment was classified by work hours (Model 2), full-time work was associated with a slower decline in cognition among retirees with lower education (β = 1.04, SE = 0.50, *p* = 0.036). In contrast, no such association was observed among higher-education retirees (*p* for interaction = 0.029). No significant interaction was found for part-time employment. Notably, in Model 1 (by work type), unpaid work among lower-educated retirees showed a positive coefficient (β = 1.17, SE = 0.71), although this was not statistically significant. Table 5 presents fully adjusted models by education level, confirming that only lower-educated full-time workers benefited, while no effect was found in the higher-education group.

**Table 4:** Regression analysis of working after retirement on the changes in cognitive function by educational levels

Models	Full Sample (*n* = 2176)		Lower Education (*n* = 957)		Higher Education (*n* = 1219)		
**Variables**	**β**	**(SE)**	**β**	**(SE)**	**β**	**(SE)**	***p*-value for interaction**
Model 1							
Working after retirement (Classification I) (ref: fully retired)							
Paid work	0.17	(0.23)	0.17	(0.39)	0.36	(0.28)	.774
Unpaid work	0.35	(0.42)	1.17	(0.71)	0.00	(0.51)	.129
Self-employment	−0.17	(0.40)	−0.11	(0.60)	−0.13	(0.55)	.966
R square	0.293		0.243		0.360		
Model 2							
Working after retirement (Classification II) (ref: fully retired)							
Full-time job	0.23	(0.29)	*1.04	(0.50)	−0.07	(0.34)	**.029**
Part-time job	0.09	(0.24)	−0.15	(0.38)	0.43	(0.29)	.220
R square	0.293		0.255		0.365		

All models were adjusted for covariates listed in Table S3. The *p*-value for the interaction tests for a significant difference in the association between education groups. Lower education: junior high school or below; Higher education: senior high school or above. *p <.05

**Table 5:** Effects of working after retirement (II) on the changes in cognitive function among retirees

Model 2	Lower education (*n* = 957)			Higher education (*n* = 1219)		
**Variables**	**β**	**(SE)**	**p value**	**β**	**(SE)**	**p value**
Baseline SLUMS score	**−0.56**	**(0.02)**	** <.001**	**−0.69**	**(0.03)**	** <.001**
Working after retirement (Classification II) (ref: fully retired)						
Full-time	**1.04**	**(0.50)**	**.036**	−0.07	(0.34)	.833
Part-time	−0.15	(0.38)	.703	0.43	(0.29)	.141
Education (ref: primary school or less)						
Junior or high school	-	-	-	-	-	-
College or more	-	-	-	-	-	-
Gender (ref: male)						
Female	0.04	(0.36)	.903	0.43	(0.23)	.060
Age (year)	**−0.88**	**(0.18)**	** <.001**	**−0.55**	**(0.10)**	** <.001**
Marital status (ref: single)						
Married	0.36	(0.37)	.334	0.14	(0.30)	.655
Economic status (ref: difficult)						
Not difficult	0.36	(0.32)	.254	0.05	(0.26)	.833
Years after retirement** (**ref: 0–5 years)						
6–9 years	0.14	(0.38)	.710	**0.55**	**(0.27)**	**.046**
More than 10 years	0.26	(0.38)	.491	0.01	(0.24)	.966
Self-rated health (ref: poor)						
Fair	0.65	(0.42)	.125	−0.05	(0.37)	.886
Excellent	0.85	(0.46)	.066	0.04	(0.37)	.905
Depression (ref: no)						
Yes	0.07	(0.47)	.878	−0.62	(0.37)	.093
Hypertension (ref: no)						
Yes	0.25	(0.31)	.419	−0.32	(0.22)	.133
Stroke (ref: no)						
Yes	−1.45	(1.16)	.176	**−2.03**	**(0.77)**	**.009**
Diabetes (ref: no)						
Yes	−0.72	(0.37)	.051	0.18	(0.29)	.542
Physical activity (ref: no)						
Yes	0.42	(0.30)	.170	−0.08	(0.21)	.714
Smoking (ref: no)						
Yes	0.30	(0.45)	.511	−0.32	(0.29)	.265
Alcohol consumption (ref: no)						
Yes	−0.18	(0.44)	.677	−0.39	(0.28)	.163
Social participation (ref: no)						
1 organization	**1.46**	**(0.37)**	** <.001**	**0.48**	**(0.23)**	**.041**
≥ 2 organizations	0.34	(0.54)	.527	**0.77**	**(0.28)**	**.007**
Pre-retirement occupations (ref: unskilled)						
Semi-skilled/skilled	0.38	(0.33)	.253	−0.38	(0.40)	.338
Managers/professionals	−0.03	(0.85)	.969	0.31	(0.41)	.452
Early retirement (ref: no)						
Yes	0.35	(0.34)	.297	**0.48**	**(0.20)**	**.020**
R square		**0.255**			**0.365**	

Supplementary analyses further tested robustness. When early retirees were excluded (Table S4), the coefficient for full-time work among lower-educated retirees remained positive but lost significance, likely due to reduced sample size. When working after retirement was reclassified as a binary indicator or modeled as a linear dose (Table S5), the interaction was no longer significant, suggesting the moderating effect of education may be specific to lower-educated retirees engaged in full-time work. Gender-stratified regressions (Table S6) indicated that working after retirement was not significantly associated with cognitive change in either men or women.

## Discussion

This study examined the associations between working after retirement and changes in cognitive function, using two complementary classifications of post-retirement work: by type (paid, unpaid, self-employment) and by hours (full-time vs. part-time). In the overall sample, neither classification showed a statistically significant association with three-year cognitive change after adjusting for covariates. However, stratified analyses revealed an important heterogeneity by education. Specifically, among retirees with lower educational attainment, full-time work was associated with significantly slower decline in cognition compared with those who were fully retired (β = 1.04, SE = 0.50, *p* = 0.036), whereas no such association was observed among higher-educated retirees. Although this finding suggests that full-time work may serve as a source of cognitive stimulation for lower-educated retirees, sensitivity analyses indicated that the interaction was not consistently significant across all model specifications. These results, therefore, highlight both the potential relevance and the limitations of interpreting educational differences in the relationship between work and cognitive aging.

### Paid or unpaid work after retirement

Prior studies suggest that paid employment may protect cognitive function by offering structured challenges, financial security, and regular social interaction. For example, Rohwedder and Willis [28] found slower declines in memory and executive function among retirees engaged in paid work, and Andel et al. [1] reported that paid post-retirement work was linked to reduced cognitive decline, particularly among socially disadvantaged groups. In the present study, retirees who engaged in paid work showed a positive, though not statistically significant, trend in cognitive change compared with those who were fully retired.

Unpaid work presents a more complex picture. Our results indicated that unpaid work was associated with a modest increase in cognitive scores overall, and the effect size was notably larger among lower-educated retirees (β = 1.17). However, this subgroup result did not reach statistical significance. This aligns with evidence that volunteer activities can promote episodic memory, working memory, and verbal fluency [31], while also reflecting the heterogeneity of unpaid roles. In Taiwan, unpaid work in retirement often involves full-time caregiving or informal consulting rather than volunteering or household chores. As Wu et al. [39] noted, such unpaid roles carry economic and cultural value—reducing childcare costs or fulfilling intergenerational responsibilities—suggesting that their cognitive implications may vary by context and social expectations.

Taken together, both paid and unpaid work after retirement appear to have potential benefits for cognitive health, though the mechanisms may differ. Paid work provides structured cognitive demands, while unpaid work—particularly in caregiving or community roles—may support cognition through social engagement and meaningful activity. Future studies should further disentangle these pathways, especially in lower-educated populations, where effect sizes in this study suggested potential benefits despite limited statistical power.

### Full- or part-time work after retirement

A growing body of research suggests that continued employment after retirement can confer cognitive and psychosocial benefits, though the magnitude may differ between full- and part-time roles. Full-time work provides frequent stimulation through structured routines, ongoing decision-making, and regular social interaction, consistent with the “use it or lose it” hypothesis [29]. Our findings support this perspective, showing that full-time employment is associated with better cognitive outcomes among retirees with lower educational attainment. This aligns with prior studies reporting that paid or full-time roles may reduce the risk of cognitive decline due to their structured cognitive demands and social connections [28, 35]. Carr et al. [6] further showed, using retirement pathway analysis (full retirement, partial retirement, and reemployment), that workers in low-complexity jobs experienced faster cognitive decline across all retirement pathways, with continued full-time work offering relatively greater protection.

However, full-time work is not universally advantageous. Demanding schedules and work-related stress may offset cognitive benefits, and prior studies have shown that high physical or psychological strain can impair memory and brain health [3, 4, 22]. This suggests that the benefits of full-time employment are conditional on job quality and individual resilience.

Part-time work may offer a more balanced alternative. Henning et al. [11] found that older adults who combined part-time work with leisure activities achieved optimal cognitive maintenance, while Kuusi et al. [18] reported that part-time workers exhibited rates of cognitive decline intermediate between those of full-time workers and fully retired individuals. Although our study did not find significant associations for part-time work, this pattern suggests that part-time employment could still support cognition, particularly when complemented by enriching social and leisure activities.

The sensitivity analysis using a linear dose specification did not yield significant results, suggesting that the relationship between work and cognition may not follow a simple dose–response pattern. Instead, our findings are consistent with a threshold effect, whereby only full-time work provides sufficient intensity of stimulation to yield measurable cognitive benefits, particularly among lower-educated retirees. Kajitani et al. [16] also argued that the “dose” of work should not be defined solely by time but must also account for job complexity, with cognitively demanding tasks playing a crucial role in sustaining cognitive function. These results also align with the ecological framework of engagement–cognition interaction, which emphasizes that cognitive maintenance is shaped by broader social and institutional contexts [34]. In Taiwan, where pension benefits for labor insurance recipients are relatively modest compared to those in the public sector, many lower-educated retirees continue to work full-time out of financial necessity rather than choice [12, 43]. Thus, the observed benefits of full-time work may reflect not only cognitive stimulation but also contextual constraints that shape opportunities for engagement.

### Education level and cognitive decline

The present study highlights the moderating role of education in the association between working after retirement and cognitive change. Given prior evidence that educational attainment shapes lifetime occupational opportunities, exposure to cognitively demanding or hazardous work, and access to stimulating environments, we treated education as a potential moderator. Education occurs before labor market entry, providing a relatively stable marker of cognitive reserve across the life course. Accordingly, we conducted stratified regressions by education level (lower vs. higher) to examine whether the cognitive implications of post-retirement work differed across educational groups.

Our findings suggest that retirees with lower educational attainment who engaged in full-time work exhibited significantly better cognitive outcomes than their fully retired peers, whereas no such association was observed among those with higher education. This pattern suggests that sustained structured engagement through employment may offer significant stimulation for individuals with limited educational resources. For lower-educated retirees, limited opportunities to accumulate reserves during formal schooling may heighten reliance on external sources of engagement, such as full-time work, to maintain cognitive function.

This interpretation aligns with the cognitive reserve framework, which posits that education and lifelong engagement build resilience against age-related decline [33]. Consistent with van Ours [38], individuals with lower education may rely more heavily on structured work activities to sustain cognition, while those with higher educational attainment may draw on a broader repertoire of cognitively enriching activities outside employment, such as social participation or lifelong learning [9, 11].

Nonetheless, the literature remains inconclusive regarding whether education influences the rate of cognitive decline. A meta-analysis by Seblova et al. [30] reported that education was strongly associated with baseline performance but not with the rate of decline. In contrast, Andel et al. [1] found that lower-educated and socially disadvantaged groups experienced steeper cognitive decline in retirement, underscoring the potential for educational disparities to amplify vulnerabilities in later life. These divergent findings highlight that the moderating role of education remains unsettled and underscore the need for further evidence from diverse contexts.

Moreover, educational disparities in cognitive aging may reflect broader structural inequalities. For example, lower-educated workers are more likely to spend their careers in physically demanding or hazardous occupations, which may increase health risks and reduce opportunities for cognitive stimulation [1, 5]. From this perspective, the apparent benefits of continued full-time work among lower-educated retirees may be shaped as much by contextual disadvantages as by cognitive reserve per se.

Finally, our sensitivity analyses revealed that the interaction between education and work hours was not consistently significant across all specifications, reinforcing the need for cautious interpretation. While the results suggest that lower-educated retirees may gain cognitive benefits from full-time post-retirement employment, these findings should be viewed as preliminary. Policy efforts should focus not only on extending employment but also on addressing educational and occupational disparities earlier in life, while promoting alternative opportunities for cognitive engagement—such as community programs and lifelong learning—for retirees across all educational backgrounds.

### General discussion: broader engagement pathways

A noteworthy feature of this study is the relatively young age composition of our cohort, with more than half of the participants aged 50–64. This contrasts with much of the existing literature on retirement and cognition, which typically focuses on adults aged 60 or older (e.g., [18, 27]). In Taiwan, however, early retirement is common due to institutional arrangements in the labor and pension systems. For example, under the Labor Insurance program, workers can begin claiming benefits as early as age 60 (with reduced payouts), and preferential retirement policies for public employees further encourage departures from the workforce before the statutory retirement age of 65 [43]. This structural context explains why a substantial share of Taiwanese retirees are still in their early 50 s or early 60 s, a stage often described as the “young-old.” Including this group provides important insights into cognitive aging not only among the oldest-old but also during earlier stages of retirement, when work-related or socially meaningful engagement is still prevalent.

Beyond employment, our findings also highlight the importance of social participation as an additional pathway to cognitive maintenance. Retirees who engaged in clubs or community organizations exhibited better cognitive outcomes, consistent with prior research linking social engagement to preserved cognitive function in later life [14, 17, 42]. Longitudinal evidence from European and Chinese cohorts further confirms that diverse social activities and supportive networks contribute to healthier cognitive aging [20, 45]. This perspective aligns with the ecological framework of engagement–cognition interaction [34], which emphasizes that cognitive health is sustained through multiple forms of meaningful engagement embedded in broader social and institutional contexts. Accordingly, strategies to promote cognitive health should not rely solely on extending employment opportunities, but also on expanding accessible and diverse avenues for social participation across the lifespan.

Another important dimension concerns gender. In Taiwan, men and women often follow different work and retirement trajectories: men are more likely to remain in formal employment until later ages, whereas women frequently retire earlier or engage in unpaid family roles such as caregiving [7]. Prior studies have suggested that such gendered trajectories may lead to differential cognitive outcomes in later life [25, 26]. To address this, we conducted gender-stratified analyses (Supplementary Table S6). Although working after retirement was not significantly associated with cognitive change in either men or women, certain covariates—such as social participation and health status—showed varying influences across genders. This underscores the importance of considering gendered pathways in retirement and post-retirement engagement when examining cognitive aging in the Taiwanese context.

### Strengths and limitations of the study

This study has several notable strengths. First, it utilized data from the longitudinal Taiwan Health and Retirement Study (THRS), which is nationally representative of retirees covered by labor and civil servant insurance, thereby enhancing the generalizability of the findings. Second, the dual-classification framework of working after retirement—by work type and work hours—provided a more nuanced analysis than prior studies that often treated post-retirement work as a single category. Third, the study explicitly examined the moderating role of education, revealing that retirees with lower educational attainment may derive greater cognitive benefits from continued full-time employment, thus contributing empirical evidence relevant to the cognitive reserve framework. Finally, cognitive function was assessed using the Saint Louis University Mental Status (SLUMS) examination, which is sensitive to mild cognitive impairment and appropriate for populations with varied educational backgrounds.

Despite these strengths, several limitations must be acknowledged. First, although we adjusted for multiple sociodemographic, health, and lifestyle covariates, endogeneity remains a concern. Employment decisions after retirement and cognitive outcomes may be simultaneously influenced by unobserved factors such as baseline cognitive ability, health status, or personality traits. Therefore, the observed associations should not be interpreted as causal. Second, the main finding—that lower-educated retirees benefited from continued full-time work—was not fully robust across sensitivity analyses. When alternative classifications of post-retirement work were applied or early retirees were excluded, the interaction between work and education was no longer statistically significant, underscoring the need for cautious interpretation. Third, data were derived from self-report surveys, which are subject to recall bias, and important factors such as genetic information, diet, hearing impairment, and leisure activities were not available. Fourth, the study examined cognitive changes over only a three-year interval, which may not capture longer-term trajectories of cognitive aging. Future research with extended follow-up and richer covariates is needed. Fifth, our key independent variable, working after retirement, was defined only at baseline (Wave 1). Potential changes in employment status during the follow-up period were not captured, which may have underestimated the dynamic nature of post-retirement work patterns. Sixth, the dataset is representative of retirees covered by labor or civil servant insurance, which includes most of Taiwan’s workforce. However, it excludes certain groups, such as farmers under separate schemes and those who never formally entered the labor force, which may limit generalizability to all older adults in Taiwan.

Finally, the policy implications of these findings should be interpreted carefully. While lower-educated retirees appeared to benefit more from continued full-time work, this may partly reflect structural inequalities in Taiwan’s pension and labor systems, where financial necessity compels disadvantaged groups to remain in the workforce. Rather than advocating extended work as a universal solution, policy efforts should prioritize reducing educational and occupational disparities, promoting equitable pension systems, and enhancing opportunities for cognitive engagement outside the labor market, such as lifelong learning, social participation, and community-based programs.

## Conclusion

This study examined the associations between working after retirement and cognitive change using nationally representative data from Taiwan. In the overall sample, neither type nor hours of post-retirement work were significantly associated with three-year cognitive trajectories. However, stratified analyses revealed that retirees with lower educational attainment who engaged in full-time work exhibited higher cognitive scores compared with their fully retired peers, whereas no such association was observed among higher-educated individuals. These findings suggest that the cognitive implications of working after retirement may differ by educational background, with full-time work potentially providing meaningful stimulation for those with fewer prior cognitive resources.

At the same time, sensitivity analyses indicated that this moderating effect was not consistently significant across all model specifications, underscoring the need for cautious interpretation. The observed associations may partly reflect contextual factors, such as financial necessity and institutional constraints within Taiwan’s pension system, rather than causal effects of work per se.

Taken together, the results contribute to ongoing debates on how post-retirement engagement relates to cognitive aging, highlighting the importance of considering heterogeneity by education. Policies aiming to promote healthy cognitive aging should therefore not rely solely on extending employment opportunities, but also support diverse forms of engagement—such as social participation and community involvement—that can provide cognitively enriching experiences across the lifespan. Future research should employ longer follow-ups and causal inference approaches to further clarify the mechanisms underlying these associations.

## Supplementary Information

Supplementary Material 1.

## Data Availability

Data can be shared upon request by the corresponding author with permission from the Health Promotion Administration, Ministry of Health and Welfare, Taiwan.
