# Supplementary material for: Different types of working after retirement on the changes in cognitive function among taiwanese retirees: 3-year follow-up study
Source: BMC Public Health. 2025 Dec 5;25:4217. doi: 10.1186/s12889-025-25325-0 (PMC12681106; doi:10.1186/s12889-025-25325-0)
Supplement: Supplementary file 1 — Supplementary Material 1. [file 12889_2025_25325_MOESM1_ESM.docx]

**Supplementary tables**

**Table S1. Types of work after retirement**

| **What is your current retirement status?** |
| --- |
| 1. Fully retired, with no part-time job or employment |
| 1. Engaged in full-time paid work |
| 1. Engaged in part-time work with irregular income |
| 1. Engaged in full-time unpaid work (e.g., serving as a consultant) |
| 1. Engaged in part-time unpaid work (excluding volunteer work) |
| 1. Assisting in a family business |
| 1. Full-time grandchild caregiving |
| 1. Other (please specify): ____________ |

**Table S2. Pre-retirement Occupational Categories**

| **What was your occupation before you retired?** |
| --- |
| 1. **Unskilled workers** |
| Homemakers, household managers, babysitters, caregivers, temporary workers, cleaners, janitorial staff, service workers, street vendors, security guards, building attendants, fishers, students, and individuals who were retired or unemployed, etc. |
| 1. **Semi-skilled workers** |
| Postal workers, drivers, shop assistants, small business owners, tailors, cooks, sales representatives, commercial agents, self-employed farmers, typists, team leaders, site supervisors, beauticians, barbers, and non-commissioned military officers, etc. |
| 1. **Skilled workers** |
| Bank staff, seafarers, administrative officers, accountants, cashiers, village chiefs, township and village representatives, actors, technicians, wholesalers, junior military officers, secretaries, legal clerks, fashion designers, kindergarten teachers, agents, contractors, police officers, firefighters, and appointed civil servants (e.g., assistant technicians, clerks, executive officers, section members), etc. |
| 1. **Professionals** |
| Elementary and secondary school principals, elementary and secondary school teachers, senior high and vocational school teachers, certified public accountants, judges, magistrates, lawyers, division chiefs in private companies, police inspectors, field-grade military officers, painters, writers, musicians, pharmacists, engineers, architects, managers, assistant managers, associate managers, deputy managers, journalists or TV reporters, city or county councilors, and civil servants at the recommended rank (e.g., senior technicians, technical specialists, secretaries), etc. |
| 1. **Senior professionals and managers** |
| University presidents, university faculty members, physicians, justices of the Constitutional Court, scientists, general-grade military officers, legislators, members of the Control Yuan, members of the Examination Yuan, National Assembly delegates, board chairpersons, chief executive officers (CEOs), and senior civil servants in specially appointed or selected ranks (e.g., ministers, bureau directors, department heads), etc. |

Table S3. Baseline Characteristics of the participants by gender (n= 2,176)

| **Variables** | **Categories** | **Total**  **(n=2,176)** | **Male (n=1,219)** | **Female (n=957)** | ***p*-value** |
| --- | --- | --- | --- | --- | --- |
| **Baseline SLUMS Score, mean (SD)** | | 23.29 (4.66) | 23.65 (4.40) | 22.83 (4.95) | **<.001** |
| **SLUMS Change Score, mean (SD)** | | −0.34 (4.64) | -0.49 (4.49) | -0.15 (4.82) | .091 |
|  |  |  |  |  |  |
| **Age** | 50-64 | 1,134 (52.1) | 586 (48.1) | 548 (57.3) | **<.001** |
|  | 65-74 | 1,042 (47.9) | 633 (51.9) | 409 (42.7) |  |
| **Education** | Primary school or less | 678 (31.2) | 305 (25.0) | 373 (39.0) | **<.001** |
|  | Junior or high school | 764 (35.1) | 433 (35.5) | 331 (34.6) |  |
|  | College or more | 734 (33.7) | 481 (39.5) | 253 (26.4) |  |
| **Marital status** | Single | 359 (16.5) | 108 (8.9) | 251 (26.2) | **<.001** |
|  | Married | 1,817 (83.5) | 1,111 (91.1) | 706 (73.8) |  |
| **Economic status** | Not difficult/ even | 1,599 (73.5) | 899 (73.8) | 700 (73.1) | .752 |
|  | Difficult | 577 (26.5) | 320 (26.2) | 257 (26.9) |  |
| **Years after retirement** | 0-5 years | 881 (40.5) | 438 (35.9) | 443 (46.3) | **<.001** |
|  | 6-9 years | 496 (22.8) | 302 (24.8) | 194 (20.3) |  |
|  | More than 10 years | 799 (36.7) | 479 (39.3) | 320 (33.4) |  |
| **Self-rated health** | Poor/not good | 291 (13.4) | 146 (12.0) | 145 (15.2) | **<.001** |
|  | Fair | 934 (42.9) | 485 (39.8) | 449 (46.9) |  |
|  | Excellent/good | 951 (43.7) | 588 (48.2) | 363 (37.9) |  |
| **Depression** | No | 1,950 (89.6) | 1,113 (91.3) | 837 (87.5) | **.004** |
|  | Yes | 226 (10.4) | 106 (8.7) | 120 (12.5) |  |
| **Hypertension** | No | 1,365 (62.7) | 736 (60.4) | 629 (65.7) | **.010** |
|  | Yes | 811 (37.3) | 483 (39.6) | 328 (34.3) |  |
| **Stroke** | No | 2,139 (98.3) | 1,191 (97.7) | 948 (99.1) | **.015** |
|  | Yes | 37 (1.7) | 28 (2.3) | 9 (0.9) |  |
| **Diabetes** | No | 1,780 (81.8) | 972 (79.8) | 808 (84.5) | **.005** |
|  | Yes | 396 (18.2) | 247 (20.3) | 149 (15.6) |  |
| **Physical activity** | No | 959 (44.1) | 500 (41.0) | 459 (48.0) | **.001** |
|  | Yes | 1,217 (55.9) | 719 (59.0) | 498 (52.0) |  |
| **Smoking** | No | 1,822 (83.8) | 895 (73.5) | 927 (96.9) | **<.001** |
|  | Yes | 354 (16.3) | 324 (26.6) | 30 (3.1) |  |
| **Alcohol consumption** | No | 1,817 (83.5) | 913 (74.9) | 904 (94.5) | **<.001** |
|  | Yes | 359 (16.5) | 306 (25.1) | 53 (5.5) |  |
| **Social participation** | No | 1,386 (63.7) | 790 (64.8) | 596 (62.3) | .456 |
|  | 1 organization | 512 (23.5) | 280 (23.0) | 232 (24.2) |  |
|  | ≥ 2 organizations | 278 (12.8) | 149 (12.2) | 129 (13.5) |  |
| **Working after retirement (Classification I)** | |  |  |  |  |
|  | Fully retired | 1,599 (73.5) | 884 (72.5) | 715 (74.7) | **.001** |
|  | Paid work | 378 (17.4) | 233 (19.1) | 145 (15.2) |  |
|  | Unpaid work | 94 (4.3) | 38 (3.1) | 56 (5.9) |  |
|  | Self-employment | 105 (4.8) | 64 (5.3) | 41 (4.3) |  |
| **Working after retirement (Classification II)** | |  |  |  |  |
|  | Fully retired | 1,599 (73.5) | 884 (72.5) | 715 (74.7) | .168 |
|  | Full-time job | 221 (10.2) | 137 (11.2) | 84 (8.8) |  |
|  | Part-time job | 356 (16.4) | 198 (16.2) | 158 (16.5) |  |
| **Pre-retirement occupations** | |  |  |  |  |
|  | Unskilled | 410 (18.8) | 125 (10.3) | 285 (29.8) | **<.001** |
|  | Semi-skilled/skilled | 1,215 (55.8) | 715 (58.7) | 500 (52.3) |  |
|  | Managers/professionals | 551 (25.3) | 379 (31.1) | 172 (18.0) |  |
| **Early retirement** | No | 1,284 (59.0) | 716 (58.7) | 568 (59.4) | .772 |
|  | Yes | 892 (41.0) | 503 (41.3) | 389 (40.6) |  |

Table S4. Sensitivity Analysis on the Interaction of Work Hours and Education, Excluding Early Retirees (n=1,284)

|  | **Model 3: By Work Hours** | | |
| --- | --- | --- | --- |
| **Variables** | **β** | **(SE)** | ***p*-value** |
| **Main Effects** |  |  |  |
| Working after retirement (Classification II) | | | |
| Full-time (ref: fully retired) | 0.30 | (0.57) | .604 |
| Part-time (ref: fully retired) | 0.55 | (0.49) | .268 |
| Lower Education (ref: Higher Education) | -0.93 | (0.42) | **.026** |
| **Interaction Terms** |  |  |  |
| Full-time × Lower Education | 0.55 | (0.78) | .486 |
| Part-time × Lower Education | -0.67 | (0.64) | .294 |
| **Key Covariates** |  |  |  |
| Baseline SLUMS Score | -0.59 | (0.03) | **<.001** |
| Age (years) | -0.68 | (0.13) | **<.001** |
| **Model Fit** |  |  |  |
| Overall *p* for Interaction (Work Hours × Ed.) |  |  | .397 |
| **R square** |  | 0.278 |  |

**Notes:**

1. **β:** regression coefficient; **SE:** Standard Error.
2. This sensitivity analysis was conducted on a subsample (n=1,284) that excluded participants who retired early, to test the robustness of the primary finding. The model was fully adjusted for all covariates as listed in Table S3.

Table S5. Sensitivity Analyses Testing the Robustness of the Work-Education Interaction (n=2,176)

|  | **Model 1:** | | |
| --- | --- | --- | --- |
| **Variables** | **β** | **(SE)** | ***p*-value** |
|  |  |  |  |
| **Part A: Analysis with a Binary Work Variable** | | | |
| Working after retirement (ref: Fully retired) | 0.13 | (0.27) | .640 |
| Working after retirement × Lower Education | 0.10 | (0.39) | .790 |
| **Part B: Analysis for a Dose-Response Relationship** | | | |
| Work Dose (Linear Trend) | -0.01 | (0.18) | .967 |
| Work Dose × Lower Education | 0.34 | (0.26) | .195 |

**Note:**

1. Both models were fully adjusted for all covariates listed in Table S3.
2. Part A uses a binary variable (Work vs. Fully Retired) to test the specificity of the finding.
3. Part B uses an ordinal 'Work Dose' variable (0=Retired, 1=Part-time, 2=Full-time) to test for a linear trend in the interaction.

Table S6. Regression Analysis of Working after Retirement (Classification II) on Cognitive Change, Stratified by Gender

|  | **Male (n=1,219)** | | | **Female (n=957)** | | |
| --- | --- | --- | --- | --- | --- | --- |
| **Variables** | **β** | **(SE)** | ***p*-value** | **β** | **(SE)** | ***p*-value** |
|  |  |  |  |  |  |  |
| **Working after retirement** **(Classification II)**(ref: fully retired) | | | |  |  |  |
| Full-time | -0.06 | (0.36) | .858 | 0.72 | (0.48) | .134 |
| Part-time | -0.03 | (0.31) | .923 | 0.22 | (0.36) | .546 |
| **Covariates** |  |  |  |  |  |  |
| **Baseline SLUMS Score** | -0.61 | (0.03) | **<.001** | -0.63 | (0.03) | **<.001** |
| **Age** (years) | -0.50 | (0.12) | **<.001** | -0.81 | (0.15) | **<.001** |
| **Education** (ref: primary school or less) |  |  |  |  |  |  |
| Junior or high school | 1.71 | (0.31) | **<.001** | 2.14 | (0.35) | **<.001** |
| College or more | 2.36 | (0.37) | **<.001** | 3.02 | (0.49) | **<.001** |
| **Marital status** (ref: single) |  |  |  |  |  |  |
| Married | 0.18 | (0.39) | .645 | -0.56 | (0.31) | .067 |
| **Economic status** (ref: difficult) |  |  |  |  |  |  |
| Not difficult | 0.14 | (0.26) | .590 | 0.38 | (0.32) | .241 |
| **Years after retirement (**ref: 0-5 years) |  |  |  |  |  |  |
| 6-9 years | 0.18 | (0.30) | .548 | 0.91 | (0.36) | **.011** |
| More than 10 years | 0.07 | (0.29) | .804 | 0.79 | (0.35) | **.023** |
| **Self-rated health** (ref: poor) |  |  |  |  |  |  |
| Fair | 0.02 | (0.37) | .957 | 0.77 | (0.41) | .061 |
| Excellent | 0.21 | (0.38) | .572 | 0.72 | (0.45) | .106 |
| **Depression** (Yes vs. No) | -0.78 | (0.41) | .055 | 0.20 | (0.43) | .650 |
| **Hypertension** (Yes vs. No) | -0.31 | (0.23) | .190 | 0.43 | (0.29) | .144 |
| **Stroke** (Yes vs. No) | -0.80 | (0.75) | .289 | -3.66 | (1.37) | **.008** |
| **Diabetes** (Yes vs. No) | -0.30 | (0.28) | .290 | -0.22 | (0.39) | .572 |
| **Physical activity** (Yes vs. No) | -0.08 | (0.23) | .731 | 0.57 | (0.27) | **.035** |
| **Smoking** (Yes vs. No) | -0.07 | (0.26) | .790 | 0.03 | (0.82) | .969 |
| **Alcohol consumption** (Yes vs. No) | -0.45 | (0.27) | .094 | 0.25 | (0.62) | .688 |
| **Social participation** (ref: no) |  |  |  |  |  |  |
| 1 organization | 0.84 | (0.27) | **.002** | 0.87 | (0.32) | **.007** |
| ≥ 2 organizations | 1.00 | (0.36) | **.005** | 0.42 | (0.41) | .303 |
| **Pre-retirement occupations** (ref: unskilled) |  |  |  |  |  |  |
| Semi-skilled/skilled | 0.40 | (0.38) | .295 | 0.13 | (0.32) | .670 |
| Managers/professionals | 0.75 | (0.46) | .107 | 0.22 | (0.52) | .669 |
| **Early retirement** (Yes vs. No) | 0.35 | (0.24) | .144 | 0.34 | (0.28) | .236 |
| **R square** |  | 0.287 |  |  | 0.318 |  |

**Note:** β: regression coefficient; SE: Standard Error. The dependent variable is the 3-year change in SLUMS score. Models were fully adjusted for all listed covariates.
